# Aging: the wound that never starts healing

**DOI:** 10.1038/s41467-025-64462-3

**Published:** 2025-09-30

**Authors:** Mikolaj Ogrodnik

**Affiliations:** 1 Ludwig Boltzmann Institute for Traumatology. The Research Center in cooperation with AUVA, Vienna, Austria; 2 Austrian Cluster for Tissue Regeneration, Vienna, Austria

**Keywords:** Medical research, Cell biology

## Abstract

Aging is a complex biological process leading to functional decline and disease susceptibility. This article proposes that chronic activation of tissue damage response mechanisms drives aging, with aged organs exhibiting features similar to those seen after acute injury, such as histolysis, inflammation, immune cell infiltration, accumulation of lipid droplets, and induction of cellular senescence. The overlap between injury and aging phenotypes is supported by evidence that interventions slowing aging often impair healing, and vice versa. This perspective offers a unifying framework to understand aging and suggests new directions for treating age-related diseases, cancer, and the aging process.

## Abstract (web summary)

Aging is a complex process resulting in a decline in organ function. Here the authors proposes that chronic activation of tissue damage response mechanisms drives aging, with aged organs displaying features similar to those seen after acute injury. This provides a unifying framework for understanding the aging process and suggests new directions for treating age-related diseases.

## Introduction

The aging process, defined by a gradual decline in organ function, remains poorly understood in terms of its underlying physiology. Our current concepts of the aging process have provided explanations for only a limited subset of the aging phenotype, resulting in treatments that extend lifespan in mammalian models by merely a few to a few dozen percent^1^. A deeper understanding of aging, not only at the molecular and cellular levels but also at the tissue level, offers an opportunity for novel concepts describing the origin and potential treatment options for the dysfunctions of aging organs. The hypothesis described here utilizes recent findings on tissue damage responses, particularly acute tissue injuries, to explain the tissue-scale phenotypes of the aging process. While the article focuses primarily on the skin, liver, and brain, most of its conclusions can be extrapolated to other organs.

### What constitutes tissue damage response?

Damage to tissues and the subsequent healing process are experienced from the earliest stages of our lives, and their effective execution enables survival. Healing is a process defined here as a response to tissue damage that aims to reduce the risks of structural disruptions and to restore tissue function. Although healing is often referred to as a single process, it is, in fact, a complex array of processes that vary based on the time elapsed since injury, the cell types investigated, and their spatial relation to the injury site^2–4^. Healing’s temporal aspects are distinct, with early stages, damage detection and response, differing significantly from the later stages that execute the repair^3^.

Healing rarely fully restores tissue function or structure, but instead trades accuracy for practical recovery within a critical timeframe. Practical recovery in this context refers to healing that allows for basic functionality and survival, even if it falls short of complete restoration. Thus, especially for mammals, it appears that timely prevention of an injury’s consequences, such as infections, re-opening of injuries or disruption of surrounding tissues’ functions, etc., is more advantageous than the accuracy of restoration. For instance, skin healing never fully restores its original structure or function^5,6^, often resulting in scarring. In contrast, a damage response that accurately restores tissue structure and function (e.g., scarless healing and regeneration of missing appendages) is more commonly observed in non-mammalian species, such as axolotls (*Ambystoma mexicanum*)^7^. However, exceptions exist even among mammals, for example, spiny mice (*Acomys*)^8^.

One of the main sources of variability in what processes are associated with an injury is the time since the induction of damage, where the initial/early stages differ substantially from the late/final stages. Canonically, healing is divided into four phases: hemostasis, inflammation, proliferation, and remodeling. While the execution of the last two phases, and thus the outcomes of healing, differ significantly between organs^9^, the phenotypes and behaviors of cells during the inflammatory phase appear to be similar (Table 1). For all cellular macrostructures, the execution of the early response to injury, the inflammatory phase, comes down to several processes: inflammation, extracellular matrix (ECM) remodeling, histolysis and tissue debridement, immune cell infiltration, lipid droplets (LDs) deposition and cellular senescence induction.

**Table 1:** Comparison of Tissue Damage Responses and Healing Processes in Selected Tissues

Organ	Inflammatory phase	Healing process
**Skin**	**- Infiltration of immune cells** **- accumulation of LDs** **- inflammation** **- induction of cellular senescence** **- histolysis and debridement** **- ECM remodeling**	**- Small and superficial injuries heal scarless, dermal injuries above certain size scar**^64^ **- Utilization of hair follicles and their stem cells**^23^
**Liver**		**- Healing reconstitutes original size without scars unless the damage response becomes chronic**^63^ **- The involvement of stem cells is most pronounced in cases of chronic injury**^28^
**Brain**		**- Involvement of neural stem cells largely ineffective**^30,100^ **- Healing of very little effectiveness; results in glia scar formation**^31^

This table provides a summary of the key features of the inflammatory phase and healing process in three organs: the skin, liver, and brain. It highlights both the commonalities observed during the inflammatory phase and the unique characteristics of each organ’s response to injury, including tissue remodeling, the role of stem cells, and the differences in regenerative capacity and scar formation among these organs.

Briefly, an injury breaks structures such as blood vessels, nerves, skeletal elements, etc., which need to be “trimmed” beyond the immediate range of an injury by processes of histolysis and debridement^4,10,11^. This is primarily done by secretion of molecules that break down tissue structures (histolysis) and a concomitant removal of damaged tissue elements and debris (debridement) by immune cells^4,10,11^. Consequently, cells and tissue structures that are even moderately damaged are degraded in order to be rebuilt at the later stages of healing. As part of histolysis, but also throughout later phases, ECM is remodeled to make tissue more suitable for angiogenesis and cell migration. This process involves a complex interplay between enzymes that degrade ECM (e.g., proteases), their inhibitors, and the deposition of ECM components like collagen. The initial damage to the tissue followed by death of cells and lysis of the ECM, as well as invasion of microbes, results in the release of damage- and pathogen-associated molecular patterns, DAMPs and PAMPs, respectively^12^. These molecules attract and activate immune cells that release pro-inflammatory molecules such as cytokines and eicosanoids. Cellular senescence, a cell cycle arrest-mediated hypersecretory phenotype, contributes to inflammation and ECM remodeling^13–16^. Changes in nutrient and oxygen availability, combined with the general shift of cellular phenotypes towards proliferation, secretion, and inflammation, result in metabolic remodeling and deposition of LDs^3,17–19^. While mechanistically it is not yet clear which signaling pathways are causal to the induction of these phenotypes, the cell cycle inhibitor p21 dramatically alters the transcriptional landscape (via RB hypophosphorylation), affecting essentially all the aforementioned processes^16,20,21^. After the clearance of damaged elements, DAMPs and PAMPs, the tissue proceeds towards the proliferation and remodeling phases^12^. Below, several examples of tissue damage responses are discussed.

#### Skin damage response

Breach of skin integrity is followed by the formation of a blood clot, which is later used as a provisional matrix to gradually replace the missing dermis^3^. Cells at the very edge of the injury die, and the edge itself undergoes histolysis and debridement, contributing to propagation of damage signals and inflammation, primarily IL-6 and TNF-α^4^. Wounding triggers rapid activation of surrounding stromal cells^2^, mobilization of underlying adipose tissue^22^ and hair follicles^23^, as well as infiltration of immune cells^10^. Not only immune, but also stromal cells become pro-inflammatory at the injury site, which at least in a short-term supports the healing process^4,16^. An effective repair of skin requires division of tasks with some cells dividing to repopulate the vacant space and others becoming senescent^2,13,16,24^. Considering that the elimination of senescent cells has been found beneficial in cases where wounds have not healed for a prolonged period^25^, it is possible that senescent cells exert different effects on healing during its various phases. Accumulation of LDs has been primarily associated with pro-inflammatory activation of immune cells^26^, but also stromal skin cells at the injury site^16^.

#### Liver damage response

While usually referred to as “regeneration”, the process of liver restoration upon injury differs from regenerative processes such as that occurring after a limb amputation of an axolotl. The differences include the lack of directionality of the regrowth; i.e., after an injury, the liver shows a directionless compensatory proliferation of all types of hepatic cells^27^. In this respect, there is a distinction in the healing pattern, where acute injuries heal by proliferation of liver cells while chronic liver injury can heal by activation of biliary epithelial stem cells^28^. If liver damage involves the removal of lobes, the following compensatory response will not regrow these lobes but will increase the size of the remaining lobes to restore the original liver-to-body mass ratio^27^. Yet, it is regeneration in a sense due to the full restoration of the organ’s size and function. From the very early stages, liver regeneration is associated with inflammation originating from DAMPs and amplified by cytokines, including IL-6 and TNF-α^19^. These attract and activate various types of immune cells that execute histolysis and debridement^10,11^, and later participate in its regeneration by growth stimulation, among others^19^. Another early response to liver damage, which is associated with regeneration, is an accumulation of LDs (often referred to as TRAS: transient regeneration-associated steatosis)^18^. The role of senescence in liver regeneration has not been thoroughly studied, and its impact on different stages of healing remains unknown. However, markers of cellular senescence have been reported in several studies following partial hepatectomy or chemical injury^14,29^.

#### Brain damage response

In comparison to the previous examples, brain is arguably the least capable of healing and provides very inaccurate restoration of its original structure upon an injury. Although injury triggers compensatory growth in neuronal progenitors, the newly generated neurons fail to integrate into the existing circuits and frequently do not survive^30^. In such conditions, glial cells dominate the damage response, leading to glial scar formation^31^. Inflammation is present from the earliest stages of the brain’s response to damage, with cytokines including TNF-α and IL-6, amongst others being reseased^32^. Inflammatory cues and disruption of the blood-brain barrier result in the activation of microglia and the infiltration of various types of immune cells, such as macrophages, neutrophils, B and T cells^33^. They participate in the histolysis involving proteases such as MMP9^34^ and together with microglia execute the subsequent debridement of damaged brain tissue^35,36^. Accumulation of LDs has been observed in the zebrafish brain after a needle injury, as well as in *post-mortem* samples from patients with traumatic brain injury^17^. Induction of cellular senescence has been shown in various types of brain injuries, between one day and several months after the insult^37^, although it is not known whether the impact of senescent cells on healing varies based on the time since injury.

While a detailed description is beyond the scope of this article, similar responses to an injury have been observed for the lungs, heart, skeletal muscle, and kidney^38–41^. Although the spatiotemporal properties of these phenotypes in relation to the injury site and healing process are unclear, their presence solidifies the notion of shared injury-related damage responses between organs.

### Aging: The wound that never starts healing

**Fig. 1:**
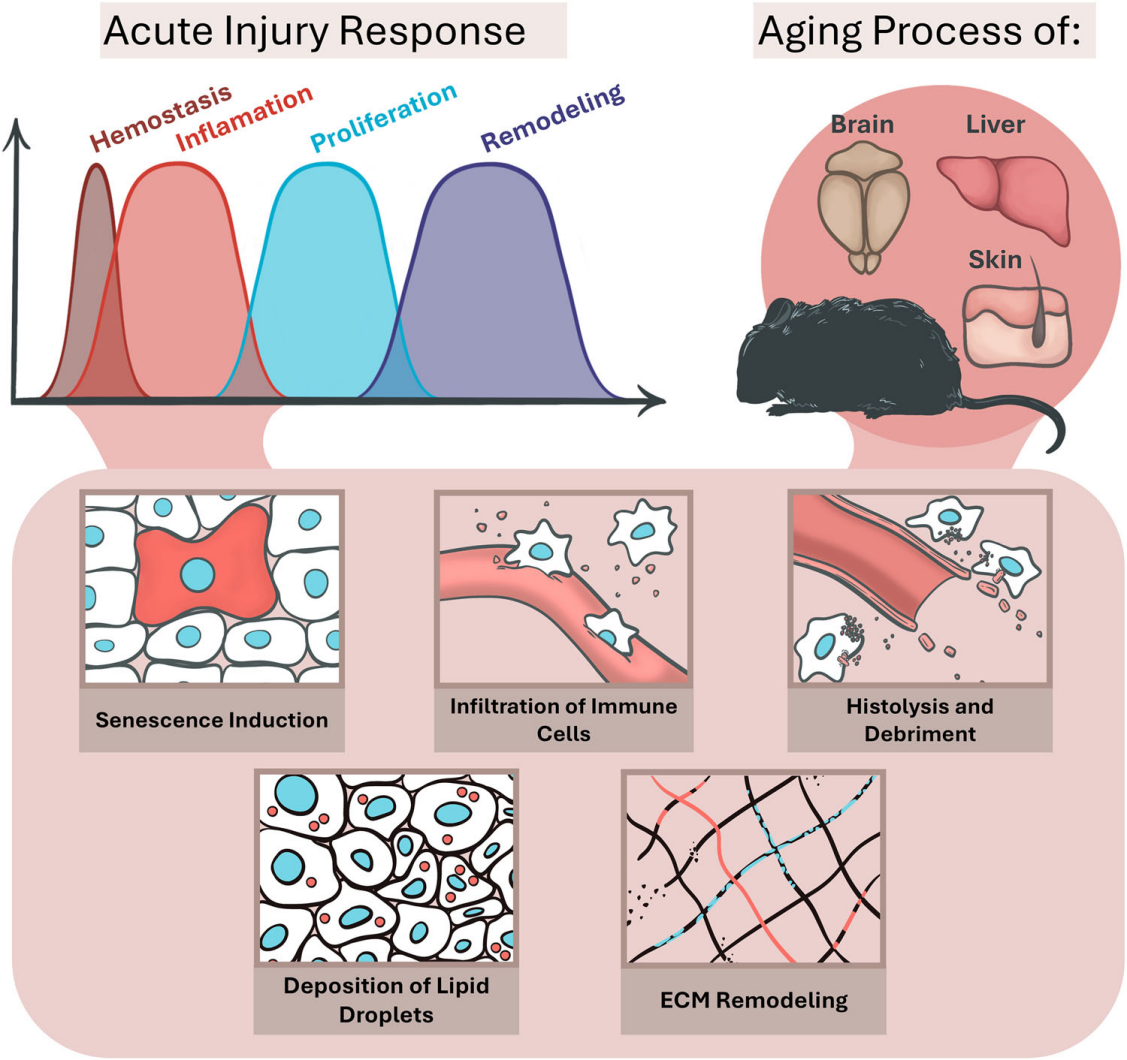
Aging organs exhibit phenotypic similarities with damage responses to acute injuries. Of the four phases of the healing process—hemostasis, inflammatory, proliferative, and remodeling—the second phase, inflammatory, exhibits tissue damage responses such as: the induction of cellular senescence, the infiltration of immune cells, inflammation, the deposition of lipids (LDs), extracellular matrix (ECM) remodeling, and histolysis. These phenotypes are also prominent in the aging process of organs such as the brain, liver, and skin.

The hypothesis posits that the aforementioned processes of how organs respond to damage are not unique to the breach of their integrity, but are also deeply entrenched within the pathologies of aging. These similarities are not accidental or unrelated; rather, organs respond to aging similarly as they would respond to an injury (Fig. 1). As the subsequent “healing” is not progressing, organs are perpetually locked in an early stage of a response to an injury. Below are several lines of evidence to support this proposition.

#### Connection of injury response to aging: molecular and cellular phenotypes

Aging leaves its signature on essentially all levels of molecular composition of cells, including the epigenome, transcriptome, and metabolome, among others^42^. While the direct comparisons of omics profiles between early responses to injuries and aging are infrequent, a recent study utilizing several types of omics investigated whether samples collected from animals and patients under stress conditions and after wounding bear resemblance to aging^43^. The study has shown that at the levels of epigenetics, transcriptomics, and metabolomics, conditions such as major surgery (and other damaging conditions) show an acceleration of the aging process (albeit in a transient fashion)^43^, indicating that indeed tissue damage responses bear an overlap with conditions of aging. Similarly, specific epigenetic changes, which closely correlate with aging, known as the epigenetic aging clock, are accelerated in conditions such as infections and various forms of tissue damage^43–45^.

On the cellular level, the phenotypes characterizing tissue damage response: inflammation, formation of LDs, infiltration of immune cells and induction of cellular senescence are directly connected to aging. Inflammation affects essentially all aging organs, to the point that it is sometimes considered the main driver of aging^46^. Interestingly, the cytokines most closely connected to aging process are IL-6 and TNF-α^47^, which are also found released upon skin, liver and brain injuries^4,19,32^. Cellular senescence, having its own contribution to inflammation^15^, is widely considered the embodiment of the aging process (as its name states) with markers of senescence found in essentially all age-related conditions investigated thus far^48^. The same goes for infiltration of immune cells, well documented for aging skin and liver^49,50^, but even immune-privileged organs, such as the brain, experience breaching of their parenchyma by immune cells like T cells^51^. Finally, accumulation of LDs has been consistently observed to increase in aging organs including the brain^52^ and liver^53^, among others^54^. While already prominent in the aging process, these injury-like cellular phenotypes are further exacerbated in numerous age-related pathologies. For example, accumulation of LDs^55^, cellular senescence^56^, infiltration of immune cells and inflammation^57^ are exacerbated in Alzheimer’s disease and non-alcoholic fatty liver disease^58^.

#### Connection of injury response to aging: morphological changes

The morphology of the aging process is usually associated with tissue atrophy^59^, which is seemingly the opposite to the healing-associated tissue growth. However, early stages of response to an injury are actually associated with histolysis and debridement, e.g., in skin^3^, liver^10^, brain^34,35^ and in amputated digits^60^, among others^9^. Similar to how the detection of damage can lead to the breakdown and subsequent replacement of structures affected by an acute injury, the detection of damage to structures affected by aging could also lead to their breakdown for a subsequent repair/replacement that in this case never comes. Thus, it is possible that in aging, ‘histolysis-like processes’ of damage response execute tissue breakdown and contribute to organ atrophy. Moreover, in many cases, aging-related decline in tissue function involves a conversion of functional tissue to fat or fibrotic tissue. Remodeling and degradation of elements of the ECM and/or deposition of fibrotic tissue is one of the most recognizable features of the aging process in organs including skin^61^, liver^58^ and brain^62^, while also being at the core of the injury response. While remodeling of the ECM occurring during aging could already provide a potential link to how aged organs behave as if they were injured, another connection is on the side of chronicity of damage perception. Damaged liver restores its mass without scarring, but if the damaging stimulus is persistent, it leads to scarring^63^. Similarly, prolonged healing of skin as in case of deep wounds exacerbates the amount of deposited collagens^64^. Therefore, it is not only the presence of aging-related damage, but also its persistence that can lead to fibrosis of aged organs. Regarding the conversion of functional tissue to fat or accumulation of LDs in aging organs^65^, it does bear similarity to the observed accumulation of LDs in injuries^16–18^. Finally, aging is also associated with a decline in certain support tissues and appendaged, for example a reduction in sub-cutaneous fat^65^ and in a number of hair follicles in skin^66,67^. As stem cells of both are known to be utilized during the healing process^22,23^, it is possible that the phenotype observed in aging is a reflection of a large-scale (non-local), healing-like response of aged skin. Accordingly, a reduction in stem cells of hair follicles during aging was directly connected to damage response of skin^68^, resembling early stages of an injury^23^.

#### Connection of injury response to aging: pathway overlaps

Disseminating the concept from the side of anti-aging interventions, one can deduce that essentially all the core pathways involved in aging have been found necessary for effectively dealing with organ injuries. In other words, if something slows down aging, it likely slows down the healing process as well. Inhibition of mTOR with rapamycin is a known intervention that alleviates aging^1^. However, it also leads to delays in wound healing^69^, disrupts proliferation and angiogenesis in models of healing^70^, and suppresses liver regeneration^71^. The Erk pathway is one of the main drivers of the injury response and the healing process that is shared between most organs^2,72^ and its inhibition was also shown to increase lifespan in a wide range of model organisms^73,74^. Similarly, caloric restriction, an intervention consistently proven to extend lifespan^1^, slows down the healing process^75,76^. Removal of senescent cells extends the time needed for closure of acute wounds^13,16^, while also extending lifespan and alleviating age-related diseases^48^. Finally, while I am not aware of evidence that depletion of the immune system slows down aging, an overactive immune system accelerates the aging process^77^ and depletion of immune cells leads to a decline in wound healing and regeneration rates^4,19^, suggesting a functional connection.

### On the molecular basis behind “injury-like” perception of aged organs

The extracellular components of our bodies are largely irreplaceable and acquire heterogeneous alterations during aging, including breaks and modifications from oxidative stress and glycation, among others^78^. Such damage could be perceived as DAMPs and lead to chronic activation of the damage response, consistent with the perception of injuries. Accordingly, DAMPs accumulate in aging organs and are of increased concentration in the bloodstream of aged individuals^79^. Alternatively or additionally, the detection of intracellular damage could lead cells to a false perception of proximity to an injury. Accordingly, we^2,16^ and others^27,37^ have observed an increase in DNA damage in cells around the injury site. The presence of intracellular damage leads to injury-associated damage responses such as activation of immune cells^77^ and induction of cellular senescence^15^. Thus, the accumulation of damage in aging cells could be perceived as proximity to an injury, leading to an injury-like damage response. Similarly to DAMPs, also PAMPs leaking through compromised barriers of aged organs could provide stimuli resembling those of an injury. Accordingly, an increase in concentration of PAMPs is known to occur during aging^80^.

### On the evolutionary basis of the “wound-like perception” of aged organs

The concept of chronic utilization of tissue damage/injury response machinery in aging aligns with evolutionary hypotheses of aging, particularly antagonistic pleiotropy (AP)^81^. The AP hypothesis, proposed by George Williams in 1957, suggests that traits beneficial early in life may have deleterious effects later, contributing to aging. The concept described here can be viewed as an example of AP, where the body’s response to damage, essential for survival and reproduction in youth, becomes maladaptive when chronically activated in later life. The shared phenotypic characteristics between injuries and aging, such as inflammation, immune cell infiltration, and cellular senescence, are crucial for survival and tissue repair early in life but may become detrimental when persistently activated during aging. This persistence might result from aging-induced damage that, while similar in nature to injury-induced damage, lacks the machinery to be properly addressed. This line of thinking aligns with another evolutionary theory of aging, proposed by Peter Medawar, that traits observed in aged organisms are beyond evolutionary selection^82^. While the hypothesis of aging viewed as the ‘wound that never starts healing’ needs to be further developed from the perspective of evolutionary biology of aging, it could provide additional useful links between results on animal physiology and the evolutionary basis of aging.

### Applying the ‘wound that never heals’ concept to develop new approaches against aging

#### New treatment options

**Fig. 2:**
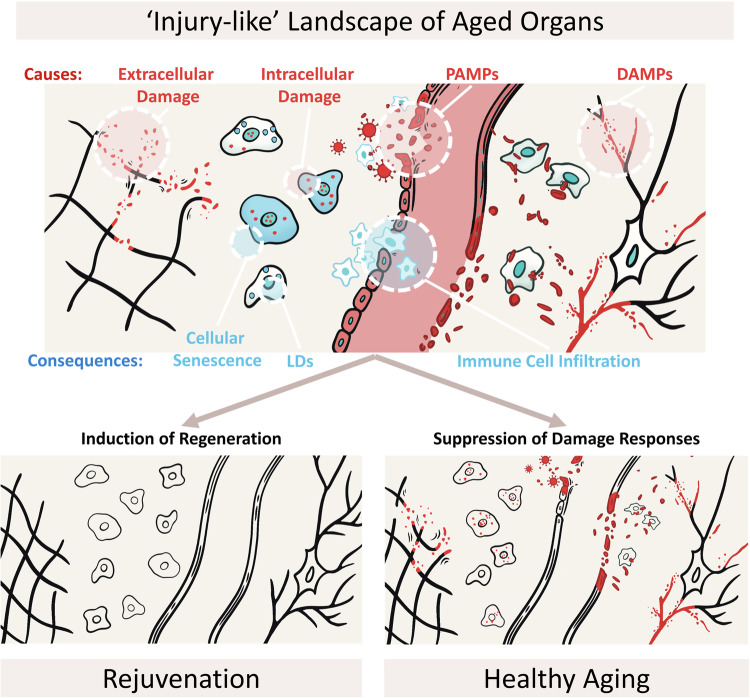
The “wound that never starts healing” landscape of aged organs and what can be done about it. Aged organs are dominated by damage responses characteristic of acute injuries (in blue), such as cellular senescence, infiltration of immune cells, and LDs deposition (and others not displayed on the figure). The causal factors behind these phenotypes could include (in red) the accumulation of extra- and intracellular damage, as well as the concentration of damage-associated molecular patterns (DAMPs) and pathogen-associated molecular patterns (PAMPs). Interventions known to alleviate the aging process silence the damage responses, leading to an alleviation of the pathologies of aging, i.e., healthy aging, but do not address the molecular causes of the ‘injury-like’ phenotypes of aged organs. In contrast, the potential effect of rejuvenation could be elicited via the induction of regeneration and a reduction of various damage types associated with aging.

As mentioned earlier, numerous treatments known to alleviate the aging process are also proven to counteract the healing process. Following this reasoning, testing substances known to slow down the healing process could reveal new means to combat aging and age-related diseases (Table 2). An obvious limitation is that new drugs are not exactly selected for their ability to slow down the rate of healing. Yet, one potential source of such compounds could originate from conditions characterized by exacerbated hallmarks of injury, such as epidermolysis bullosa or psoriasis. In fact, a few drugs commonly used against these conditions, e.g., adalimumab or apremilast, have shown promise against certain age-related conditions^83,84^, suggesting that more such treatments could treat the consequences of aging. Another approach to developing anti-aging compounds could involve countering damage responses. While there is already a prominent advancement in development of therapies against inflammation^46^ and cellular senescence^15^, more emphasis could be put on other damage-associated tissue features such as histolysis, DAMPs and PAMPs. Accordingly, the recent advancement in inhibiting TLR and NLRP3 receptors has alleviated certain age-related conditions^79,80^. Hypothesizing that pathologies of aging are due to damage responses triggered in aged organs, silencing these responses may not “cure aging” but could alleviate age-related diseases, thereby leading to healthier and potentially delayed aging (Fig. 2). Undoubtedly, inhibiting processes involved in healing raises concerns about a reduced tissue regeneration capacity. Strategies to mitigate these effects could involve short-duration treatments or optimal dosing (such as in studies with rapamycin or fasting^85^), as it might be possible to achieve a dose that inhibits early tissue damage responses without significantly impacting subsequent healing processes. Regarding the timeframe, intermittent regimens such as those used for the one-two punch application of senolytics in cancer^86^ could also minimize adverse effects.

**Table 2:** Experimental approaches aligned with “Aging: The Wound That Never Starts Healing” hypothesis to combat aging process

Research Direction	Treatment Options	Potential benefits
**Inhibition of Wound Healing Processes**	**Drugs used for epidermolysis bullosa or psoriasis, e.g., adalimumab, apremilast**^83,84^**, among others**	**Reduction in histological markers of aging that are associated with tissue damage responses; alleviation of age-related diseases; potential slowdown of the aging process**
**Damage Response Inhibition**	**Therapies against inflammatory responses and cellular senescence**^15,46^	
**DAMP and PAMP Reduction**	**Inhibitors of receptors for PAMPS and DAMPs such as TLR and NLRP3**^79,80^	
**Regeneration Induction**	**Secretory factors known to be driving epimorphic regeneration, e.g., FGF and BMP family members**^95,96^	**Reduction in factors underlying tissue damage responses such as intra- and extra-cellular damage; rejuvenation and negligible senescence**
**Cellular Reprogramming**	**Pharmacological or pharmacogenetic forced dedifferentiation of cells**^98^	

This table summarizes key research directions focused on inhibiting ‘injury-like’ phenotypes in aging organs, along with exemplary corresponding treatment options.to enhance tissue repair and regeneration processes.

#### Connection to cancer

Cancer is one of the most common age-related pathologies, with a risk of occurring in essentially all cellular tissue structures. Referred to as the “wound that never heals”^87,88^, cancer often occurs at the site of an injury or as a consequence of the healing process^89,90^. The common features of healing and cancer include elevated proliferation, migration, and angiogenesis, and the detailed characteristics of this overlap have been recently reviewed^91^. Our research results further support this concept, showing that the spatial range of healing can be visualized as a lump of tissue mass surrounding the wound, which promotes proliferation and angiogenesis, among others, resembling the characteristics of a tumor^2^.

It is necessary to stress that the aging process is the main risk factor for cancer across tissues^92^. At the same time, cancer exhibits features that are often opposite to aging, which is burdened with tissue atrophy and loss of cells. Thus, going back to the phases of the healing process, the behavior of cancer matches the proliferation phase, while aging corresponds better to the inflammatory phase. One question this hypothesis leads to is whether aging-induced cancer is another façade of the “injury-like” appearance of aged organs. The immediate next question is whether cancer results from an attempt to progress with the healing process, moving from the inflammatory to proliferation phases. As anti-aging treatments (reducing “injury-like” properties) reduce cancer risk^93,94^, it is intriguing to speculate that dampening the damage response in aged organs may prevent the transition from the inflammatory to the proliferative phase. At the same time, abolishing the induction of injury-associated responses such as immune cell infiltration or cellular senescence could lead to cancer. Consistently, mice deprived of a functional immune system or the machinery responsible for senescence induction show an increased risk of cancer development^91^. In summary, the “wound that never stops healing” phenotype of cancer could represent a dysregulated process of dealing with damage responses induced in aged organs, thus attempts to transition aged organs from an inflammatory to a proliferative phase could potentially lead to cancer.

#### Inducing regeneration instead of healing

Regeneration is a relatively poorly understood process of accurately reconstituting damaged tissues. While regeneration shares at least some of the tissue damage responses, such as inflammation, infiltration of immune cells, and cellular senescence^24^, it differs in how these responses transition to reconstitute the damaged region. Arguably, the component that distinguishes regeneration from healing is the ability to re-specialize existing cells towards replacing the missing ones. This can be done either directly, through re-specialization of existing cells (followed by compensatory growth), as in morphallaxis of *hydra*, or indirectly, through de-specialization of cells into pluripotent cells and formation of so-called ‘blastema,’ as in epimorphic regeneration of an axolotl^7^. While the inter-species differences seem insurmountable, such processes are possible in mammals^8^ and can be induced artificially to a limited extent^95,96^. For the latter, one approach is to introduce secretory factors such as members of the FGF and BMP family, which change the trajectories of damage responses from healing to regeneration^95,96^. Another approach is to force de-specialization of cells using methods such as reprogramming^97^. While to my knowledge, the former has not been tested against aging, the latter has shown some promise against aging-associated conditions^98^. Hypothetically, the induction of regeneration should remove the damage that underlies the inflammatory phase of healing and, consequently, the phenotypes of the damage responses thus delaying or even preventing aging process (Fig. 2). This hypothesis is consistent with organisms having a profound capacity to regenerate, showing negligible aging^99^. In summary, the hypothesis presented here defines aging as a progressive form of tissue injury and proposes a set of interventions aimed at alleviating aging (by suppressing damage responses) or treating aging (by resolving these responses and regenerating tissues).

### Conclusions

Here, it is argued that during aging, organs become locked in the early stages of the “healing process” that phenotypically correspond to the inflammatory phase. The damage responses activated within this state include the induction of cellular senescence, inflammation, LDs deposition, ECM remodeling, histolysis, and the infiltration of immune cells. Possibly, the “injury-like” perception of tissues undergoing aging originates from accumulated damage or its immediate consequences, such as DAMPs and PAMPs. While existing anti-aging interventions can, to a certain extent, suppress some of the damage responses, the concept proposed here might provide additional research avenues for further advancement. Ideally, the aging research of the immediate future will go beyond silencing the damage responses to the injury-like state of aged organs and towards addressing the roots of aging through regeneration.
